# A Chinese family with Noonan syndrome caused by a heterozygous variant *in LZTR1*: a case report and literature review

**DOI:** 10.1186/s12902-020-00666-6

**Published:** 2021-01-06

**Authors:** Xiu Zhao, Zhuoguang Li, Li Wang, Zhangzhang Lan, Feifei Lin, Wenyong Zhang, Zhe Su

**Affiliations:** 1 Endocrinology Department, Shenzhen Children’s Hospital, 7019# Yitian Road, Futian District, Shenzhen, 518038 Guangdong Province China; 2 School of Medicine, Southern University of Science and Technology, Shenzhen, 518055 Guangdong Province China; 3 Radiology Department, Shenzhen Children’s Hospital, Shenzhen, 518038 China

**Keywords:** Noonan syndrome, *LZTR1*, Autosomal dominant, Growth hormone deficiency

## Abstract

**Background:**

Noonan syndrome is an inherited disease involving multiple systems. More than 15 related genes have been discovered, among which *LZTR1* was discovered recently. However, the pathogenesis and inheritance pattern of *LZTR1* in Noonan syndrome have not yet been elucidated.

**Case presentation:**

We herein describe a family with *LZTR1-*related Noonan syndrome. In our study, the proband, sister, mother, maternal aunt and grandmother and female cousin showed the typical or atypical features of Noonan syndrome. Only 3 patients underwent the whole-exome sequencing analysis and results showed that the proband as well as her sister inherited the same heterozygous *LZTR1* variant (c.1149 + 1G > T) from their affected mother. Moreover, the proband accompanied by growth hormone deficiency without other associated variants.

**Conclusion:**

In a Chinese family with Noonan syndrome, we find that the c.1149 + 1G > T variant in *LZTR1* gene shows a different autosomal dominant inheritance from previous reports, which changes our understanding of its inheritance and improves our understanding of Noonan syndrome.

## Background

Noonan syndrome (OMIM 163950), with an estimated incidence of every 1000–2500 live births, is an autosomal dominant (AD) or recessive (AR) disorder that involves multiple systems with high heterogeneity [1]. To date, more than 15 genes associated with Noonan syndrome have been reported [2, 3], among which, variants of *LZTR1* have been newly associated with the etiology of Noonan syndrome since 2014 [4–6]. *LZTR1* (OMIM 600574) is the abbreviation for the leucine zipper-like transcriptional regulator 1 gene and is located on 22q11.2. So far, less than 50 cases of Noonan syndrome have been associated with *LZTR1* variants [2, 4, 5, 7–10], making its genetic pattern not well understood. For example, the c.1149 + 1G > T variant in *LZTR1* gene was characterized by AR inheritance in the previous literature involving 3 patients with Noonan syndrome [7, 8, 11]. However, in our patients, we find that the facts are not exactly as reported in the previous literature. Here we report a Chinese family with Noonan Syndrome caused by a heterozygous variant in *LZTR1*, which will change the previous understanding of *LZTR1* inheritance and improve our understanding of Noonan Syndrome.

## Methods and materials

### Clinical reports

**Fig. 1:**
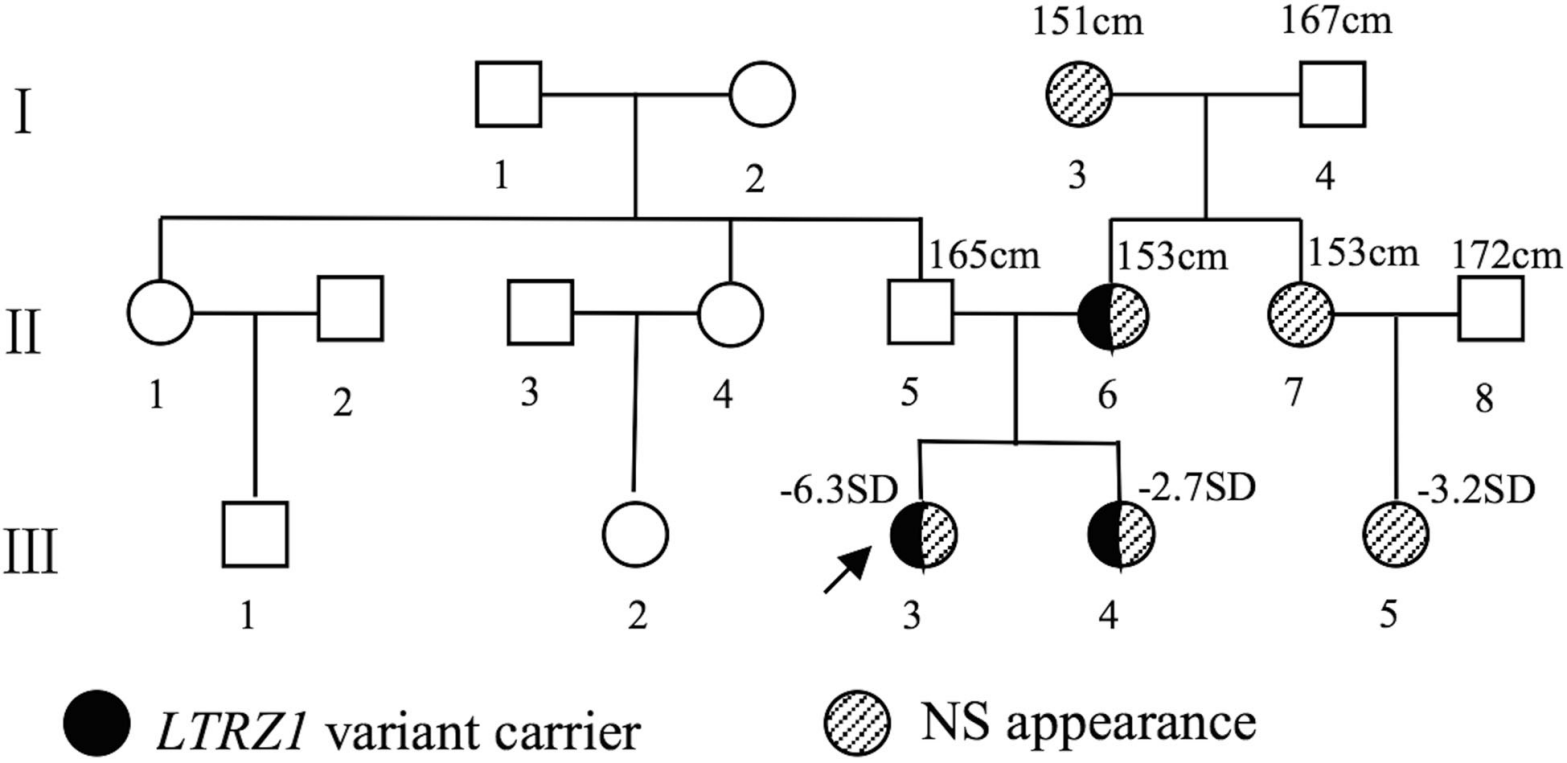
Pedigrees of the Noonan syndrome families with variant c.1149 + 1G > T of the *LZTR1* gene. The arrow indicates the proband. Black indicates that the patient exhibited variant c.1149 + 1G > T of *LZTR1*. Stripes indicate that the patient had a NS-like appearance to varying degrees. The numbers above the patients indicates their height.

**Patient 1** The proband (Fig. 1, III-3), a 6.6-year-old girl, was admitted to our hospital because of short stature. As the first child of nonconsanguineous parents, she was born at 41 weeks of gestation via vaginal delivery, whose birth weight and length were 1800 g (<P3^rd^) and 47 cm (<P3^rd^), respectively. She showed significant growth retardation since the newborn stage, and now her height, weight and body mass index (BMI) were 90.5 m (− 6.3 SD), 11 kg (− 4.9 SD) and 13.4 kg/m^2^ (P10-25th), respectively. Her ratios of arm span/height and sitting height/height were 0.92 (− 2 SD) and 0.58 (+ 3 SD) according to the standard reference values [12]. Her psychomotor development was mildly delayed.

**Fig. 2:**
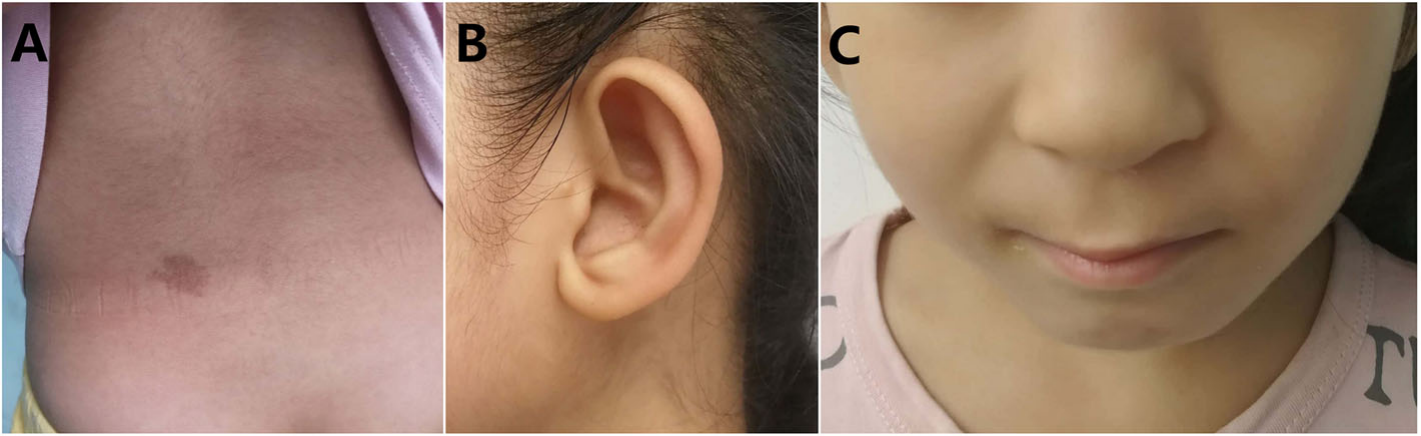
Clinical images showing Noonan-like features in the proband, who provided consents. **a** Café au lait spots and mild hypertrichosis. **b** Low-set, oval-shaped, posteriorly rotated ears with a thick helix. **c** Short broad nose with a depressed root and full tip, deeply grooved and long philtrum, and high and wide peaks of the vermilion

Physical examination: She showed the following typical features of Noonan syndrome (Fig. 2): hypertelorism; downslanting palpebral fissures; epicanthal folds; low-set, oval-shaped, posteriorly rotated ears with a thick helix; a short broad nose with a depressed root and full tip; a deeply grooved and long philtrum; high and wide peaks of the vermilion; a highly arched palate; micrognathia; a short neck; cubitus valgus; scoliosis; café au lait spots; and mild hypertrichosis. She also presented with squinting, refractive errors and nystagmus.

Auxiliary examination: The IGF-1 level was 61 μg/L (− 1.9 SD) and growth hormone (GH) stimulation test (insulin and levodopa stimulation test) showed that GH peak was 5.54 ng/mL (cut-off value: < 7 ng/mL). The electrocardiogram test showed frequent premature ventricular beats (the second and third rhythms), X-rays and MRI of the spine showed hemivertebral deformity of the third thoracic vertebra and scoliosis (Cobb’s angle = 28°), and her bone age was 5.5 years old. In addition, hormone levels of the adrenal gland, thyroid gland and gonad were normal; the tumor markers (includes AFP, HCG and CEA) were also negative. No abnormalities were found during the ultrasound examination of the heart, liver, kidneys, uterus or ovaries, so did the MRI of the craniocerebrum. Besides, her karyotype was 46, XX.

According to the clinical manifestation and laboratory tests, she was diagnosed as Noonan syndrome with GH deficiency clinically [13]. Due to her serious scoliosis and hemivertebral deformity, recombined human GH treatment was not recommended.

**Fig. 3:**
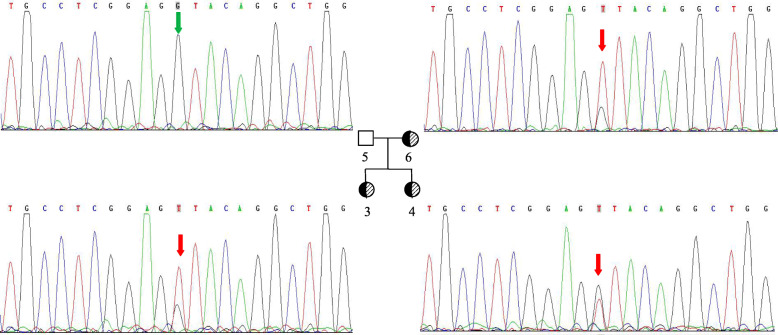
Sanger validation of mutation c.1149 + 1G > T of the *LZTR1* gene in the proband’s family. Variant c.1149 + 1G > T is shown in red. Absence of variant c.1149 + 1G > T is shown in green

**Patient 2** (Fig. 1, III-4) was the younger sister of the proband. Her birth weight and height were 3000 g (P50th) and 49 cm (P25th), respectively, with a gestational age of 38 weeks. Her height and weight were 82.5 cm (− 2.7 SD) and 10.7 kg (− 1.8 SD) at the age of 2.5 years. She had similar facial appearance to patient 1. She had no skeletal abnormities except for pectus carinatum. She didn’t show mental retardation, nor did she have any diseases of the heart and genitourinary system. The patient was also diagnosed as Noonan syndrome clinically (Fig. 3).

**Suspected patient 3** (Fig. 1, II-6) was the mother of patients 1 and 2. She was 27 years old, and her height was 153 cm (− 1.7 SD). She showed the mild phenotype of Noonan syndrome: hypertelorism; downslanting palpebral fissures; low-set, oval-shaped, posteriorly rotated ears with a thick helix; a highly arched palate; and prominent nasolabial folds. She didn’t show mental retardation, nor did she have any diseases of the heart, genitourinary system and skeletal system (Fig. 3).

**Suspected patient 4** (Fig. 1, I-3) and **suspected patient 5** (Fig. 1, II-7) were the mother and younger sister of patient 3, respectively. They had appearance features similar to those of patient 3. Their heights were 151 cm (− 1.9 SD) and 153 cm (− 1.7 SD), respectively.

**Suspected patient 6** (Fig. 1, III-5) was the daughter of patient 5. At the age of 3 years, she showed a short stature (84.3 cm, − 3.2 SD) and similar facial appearance to patients 1. She didn’t show mental retardation, nor did she have any diseases of the heart, genitourinary system and skeletal system.

### Whole exome sequencing

After obtaining the informed consent from her family, whole exome sequencing (WES) analysis was performed on the proband and her family. Genomic library was built using a standard library construction kit, and exons were captured using the target sequence capture probe. All of the exons (including the 50 bp flanking piece on either side) were captured in a single reaction, and genes related to the RASopathies were thus considered. ﻿The average sequencing depth, average coverage and 10X coverage (coverage of sites with depth greater than 10) in the target region were 153.02X, 99.43 and 96.28%, respectively. A standard bioinformatics pipeline was utilized for variant identification with the help of Genome Analysis Toolkit (GATK) [14] software following the best practice guidelines recommended by the GATK [15, 16]. Candidate variants were retained as follows: (1) rare variants with a minor allele frequency of < 1% in the ExAC, dbSNP, 1000 Genomes, gnomAD and local databases, and (2) functional variants including frameshift, splice, nonsense, missense and synonymous variants that can affect splicing. Then, we utilized a hypothesis-free approach to analyze all of the phenotype-related genes. Nonetheless, exome sequencing is limited in detecting large deletions/duplications and deep intronic variants.

Eventually, we identified a heterozygous variant, c.1149 + 1G > T, under accession number NM_006767.3 in the *LZTR1* gene. No other variants were detected in the *LZTR1* gene or in the other RASopathy genes in the proband. The proband did not harbor other mutations that have been associated with Noonan syndrome, GH deficiency, skeletal dysplasia and other genetic diseases. The splicing variant was ranked as “likely pathogenic” according to the 2015-ACMG Standards and Guidelines [17]. Sanger sequencing showed that the variant of the proband and her sister was inherited from the proband’s mother, but her father without Noonan syndrome didn’t carry the variant (Fig. 3). Unfortunately, patients 4–6 rejected the genetic analysis, and patients 2–6 didn’t agree to share their photos.

## Discussion and conclusion

Noonan syndrome is a genetic disease involving multiple systems, but as many as 25% of patients cannot get a clear genetic diagnosis, so its clinical diagnosis is equally important. The phenotype of Noonan syndrome is variable, including: short stature, congenital heart defects and/or cardiomyopathy, characteristic craniofacial dysmorphism and childhood benign or malignant tumors (such as leukemia and solid tumors). The diagnosis of Noonan syndrome mainly depends on its typical clinical manifestations [1, 18] The typical feature of Noonan syndrome is short stature, but some patients with Noonan syndrome have GH deficiency, as previous studies reported, 3 patients with Noonan syndrome have been diagnosed with GH deficiency [7, 8, 19]. In our study, the proband (III-3) was the fourth Noonan syndrome patient identified as having GH deficiency (Table 1).

**Table 1:** *LZTR1*-related Patients with Noonan syndrome and GH deficiency in the literature

Literature	Age	Sex	Hereditary form	Variant location	Nucleotide change	Amino acid change	Origin of variant	Phenotype			
	(years)							Facial and physical features	Short stature	Cardiac defect	Others
Johnston, J. J, et al. [8]	3.2	F	AR	Within intron 16 of *LZTR1* affecting BTB 2	c.1943–256C > T	*70G > A	MC, FC	Prenatal polyhydramnios; proptosis; ptosis; wide mouth; low-set ears; bulbous nasal tip; relative macro- cephaly	+	HCM; small ASD	Delayed development; decreased muscle mass and motor coordination
Nakaguma, M. A, et al. [19]	12.5	M	AR	Kelch 5	c.881G > T	p.R294L	MC	Ptosis; triangular face; high-arched palate; low-set ears; micrognathia; pectus excavatum	+	Transposition of the great vessels; PVS; inter- ventricular and interatrial communication	NA
				BTB 2	c.2212C > T	p.Q738*	FC				
Pagnamenta, A. T, et al. [7]	5	M	AD	Kelch 2	c.407A > G	p.Y136C	De novo	Congenital ptosis; depressed nasal bridge; low-set, posteriorly rotated ears; pointed chin; wide intermamillary distance; barrel-shaped chest; pectus excavatum; 2–3 toe syndactyly; cryptorchidism	+	Mild PVS	Delayed speech and language development; generalized hypotonia; delayed development
Our study	6.6	F	AD	Kelch 6 and BTB might be lost	c.1149 + 1G > A	Disrupts splice site (donor)	MC	Hypertelorism; downslanting palpebral fissures; epicanthal folds; squinting; nystagmus; low-set, oval-shaped, posteriorly rotated ears with a thick helix; short broad nose with a depressed root and full tip; deeply grooved and long philtrum; high and wide peaks of the vermilion; highly arched palate; micrognathia; short neck; cubitus valgus; scoliosis; pectus excavatum; café au lait spots; mild hypertrichosis	+	–	Delayed psychomotor development; hemivertebra deformity; refractive errors

*F* female, *M* male, *AD* autosomal dominant, *AR* autosomal recessive, *FC* farther carrier, *MC* mother carrier; −: feature absent; +: feature present; *NA* not applicable, *HCM* hypertrophic cardiomyopathy, *PVS* pulmonary valve stenosis, *ASD* atrial septal defect

To make accurate diagnoses quickly and effectively, we performed WES for molecular diagnoses and results showed that there was a heterozygous variant (c.1149 + 1G > T) in the *LZTR1*. Based on variant c.1149 + 1G > T in the *LZTR1* gene segregating with Noonan syndrome-related phenotype in multiple affected family members, we speculated that the pedigree presented as dominant inheritance. Previous studies have demonstrated that *LZTR1* variants can be acquired via AR or AD inheritance [2, 4, 7, 8, 20]. Variant c.1149 + 1G > T of *LZTR1* gene was used to be reported as compound heterozygous variants in three patients with Noonan syndrome [7, 8, 11] (Table 2). Our patients had the Noonan syndrome phenotype and the heterozygous variant inherited in the AD form. Additionally, the phenotype of Noonan syndrome ranges widely, from a normal appearance to typical features of Noonan syndrome. Families with AD NS exhibited vertical transmission of the phenotype with differential penetrance. Therefore, our report displayed the AD mode of hereditary Noonan syndrome with incomplete penetrance.

**Table 2:** Patients with Noonan syndrome with variant c.1149 + 1G > T of *LZTR1* in the literature

Literature	Age (years)	Sex	Hereditary form	Variant location	Nucleotide change	Amino acid change	Origin of variant	Phenotype				
								Facial and physical features	Short stature	Cardiac defect	ECG	Others
Perin, F, et al. [11]	4	M	AR	Kelch 6 and BTB might be lost	c.1084C > T	p.R362*	MC	Broad forehead; hypertelorism; downward-slanting palpebral fissures; posteriorly rotated ears with a thickened helix; broad thorax with a webbed neck	NA	Severe HCM; mild PVS	Broad QRS complexes; RBBB; left axis deviation	NA
				Kelch 6 and BTB might be lost	c.1149 + 1G > T	Disrupts splice site	FC					
Pagnamenta, A. T, et al. [7]	6.8	M	AR	BTB2	c.2062C > T	p.R688C	De novo	Blue irides; downslanting palpebral fissures; convergent squinting; ptosis; hypertelorism; low-set, posteriorly rotated ears; pectus carinatum; wide neck; joint hypermobility; square thumb	+	NA	NA	Mild developmental delay and delayed speech and language development; hypermetropia; hyperacusis; hypotonia
				Kelch 6 and BTB might be lost	c.1149 + 1G > T	Disrupts splice site						
Johnston, J. J, et al. [8]	2.1	F	AR	Kelch 1–6 and BTB might be lost	c.27delG	p.Q10fs	FC	Prenatal polyhydramnios; depressed, broad nasal bridge; relative macrocephaly; nevus flammeus on forehead; midface retrusion with marked frontal bossing; high anterior hairline; nevus flammeus on forehead; downslanted palpebral fissures; bilateral epicanthus with widely spaced eyes; long philtrum; full, sagging cheeks; short neck; broad chest; relatively short arms and legs	NA	Levocardia; small ASD; patent foramen ovale	Fetal bradycardia	Hypotonic; Intestinal malrotation
				Kelch 6 and BTB might be lost	c.1149 + 1G > A	Disrupts splice site	MC					
our study	6.6	F	AD	Kelch 6 and BTB might be lost	c.1149 + 1G > A	Disrupts splice site (donor)	MC	Hypertelorism; downslanting palpebral fissures; squinting; nystagmus; epicanthal folds; low-set, oval-shaped, posteriorly rotated ears, thick helix; short broad nose with a depressed root and full tip; deeply grooved and long philtrum; high and wide peaks of the vermilion; highly arched palate; micrognathia; short neck; cubitus valgus; scoliosis; pectus excavatum; café au lait spots; mild hypertrichosis	+	–	Frequent premature ventricular beats	Delayed psychomotor development; hemivertebra deformity; refractive errors
	2.5	F	AD	Kelch 6 and BTB might be lost	c.1149 + 1G > A	Disrupts splice site (donor)	MC	hypertelorism; downslanting palpebral fissures; epicanthal folds; low-set, oval-shaped, posteriorly rotated ears with a thick helix; short broad nose with a depressed root and full tip; deeply grooved philtrum; high and wide peaks of the vermilion; highly arched palate; micrognathia; short neck; and pectus carinatum	+	–	–	–
	27	F	AD	Kelch 6 and BTB might be lost	c.1149 + 1G > A	Disrupts splice site (donor)	MC	hypertelorism; downslanting palpebral fissures; low-set, oval-shaped, posteriorly rotated ears with a thick helix; highly arched palate; and prominent nasolabial folds	–	–	–	–

*F* female, *M* male, *AD* autosomal dominant, *AR* autosomal recessive; −: feature absent; +: feature present; *NA* not applicable, *FC* farther carrier, *MC* mother carrier, *HCM* hypertrophic cardiomyopathy, *PVS* pulmonary valve stenosis, *ASD* atrial septal defect, *RBBB* right bundle branch block

As we know, more than 15 gene variants are known to be involved in the etiology of Noonan syndrome. Pathogenic variants in the genes encoding proteins implicated in the RAS-MAPK signaling pathway are responsible for Noonan syndrome. These gene variants function upstream of the RAS/MAPK cascade or its regulation and they dysregulate the RAS/MAPK pathway, leading to sustained or excessive activation of ERK (which defines RASopathies) [1]. *LZTR1*-related Noonan syndrome was recently described. *LZTR1* is a highly conserved gene and encodes a protein characterized by six tandemly arranged Kelch motifs at the *N*-terminus and two BTB/POZ (broad complex, tramtrack and bric-a-brac/Pox virus and zinc finger) domains at the *C*-terminus. *LZTR1* is an important regulator of the normal cell cycle and acts as a tumor suppressor. Additionally, *LZTR1* has been found to be a conserved regulator of RAS ubiquitination and signaling [20–23].

In the current study, the variants of *LZTR1* associated with Noonan syndrome were located in both the Kelch and BTB/POZ domains, and AD Noonan syndrome has been attributed to the Kelch motifs, especially Kelch motifs 1–4 [10, 20, 23]. A new study showed that more than one RVxF motif is located between Kelch 5 and Kelch 6 in the *LZTR1* gene. RVxF is a binding location of the protein phosphatase-1 (PP1) [23, 24]. Variant c.1149 + 1G > T is located in Kelch domains 5–6. This variant can cause splice abnormalities and produce truncated proteins and thus might influence the binding function of the RVxF motif and PP1. To our knowledge, more than 50% of phosphoserine/threonine dephosphorylation reactions are catalyzed by PP1 in mammalian cells [25]. PP1 multifunctionally interacts with dozens of polypeptides that function as substrates, inhibitors, chaperones, anchoring/scaffolding proteins, and substrate-specifiers [24, 26] and even those associated with heart physiology [27]. The proband’s arrhythmia could be associated with the dysfunction of PP1.

In conclusion, we have described a rare condition of Noonan syndrome, caused by a heterozygous variant (c.1149 + 1G > T) in *LZTR1*, manifested as autosomal dominant inheritance, which is different from previous reports, which changes our understanding of the inheritance of *LZTR1* gene and improves our understanding of Noonan syndrome. Besides, we find that patients with Noonan syndrome may suffer GH deficiency at the same time, which will help us to enrich the clinical spectrum of Noonan syndrome. Patients with Noonan syndrome should be tested for possible GH deficiency coincidence.

## Data Availability

The dataset analyzed in the current study is available from the corresponding author upon reasonable request.

## Abbreviations

AD: Aautosomal dominant
AR: Autosomal recessive
GH: Growth hormone
*LZTR1*: Leucine zipper-like transcriptional regulator 1
PP1: Protein phosphatase-1
